# Clinical Trial: Study to Investigate the Efficacy and Safety of the Alpha‐2‐Delta Ligand PD‐217,014 in Patients With Irritable Bowel Syndrome

**DOI:** 10.1111/apt.18487

**Published:** 2025-01-15

**Authors:** Lesley A. Houghton, Simiao Gao, Steven A. Gilbert, Benoit Coffin, Magnus Simren, Jeremy D. Gale, the A4451007 study investigators

**Affiliations:** ^1^ Division of Gastroenterology and Surgical Sciences Leeds Institute of Medical Research, University of Leeds Leeds UK; ^2^ Division of Gastroenterology and Hepatology Mayo Clinic Jacksonville Florida USA; ^3^ Global Biometrics and Data Management Pfizer Inc Cambridge Massachusetts USA; ^4^ Service d'Hépato‐Gastro‐Entérologie, Assistance Publique‐Hôpitaux de Paris Hôpital Louis Mourier, DMU ESPRIT‐GHU AP‐HP‐Nord Colombes France; ^5^ Department of Molecular and Clinical Medicine Institute of Medicine, Sahlgrenska Academy, University of Gothenburg Gothenburg Sweden; ^6^ Inflammation and Immunology Research Unit Pfizer Inc Cambridge Massachusetts USA

**Keywords:** alpha‐2‐delta ligands, irritable bowel syndrome, PD‐217,014

## Abstract

**Introduction:**

Despite the emergence of drugs to treat irritable bowel syndrome (IBS), improving abdominal pain can still be challenging. α_2_δ ligands, such as gabapentin and pregabalin, are sometimes used off‐label to tackle this problem. However, evidence for efficacy is limited, and no large‐scale studies have been published.

**Aim:**

To study the efficacy of the α_2_δ ligand PD‐217,014 in IBS.

**Methods:**

This multi‐centre, double‐blind, randomised, placebo‐controlled, parallel group study randomised participants with Rome II‐defined IBS to 150 or 300 mg b.d. of PD‐217,014 or placebo b.d. for 4 weeks. The primary efficacy endpoint was responder, defined as having adequate relief of abdominal pain/discomfort for ≥ 50% of the active treatment period. Key secondary endpoints were change from baseline in abdominal pain, bloating, stool frequency/consistency, and global assessment of IBS symptoms.

**Results:**

We randomised 330 participants [aged 19–73 years; 209 (65%) female] satisfying Rome II criteria, 322 (98%) were treated, and of whom 271 (84%) completed the study. In this study, 321 satisfied Rome IV criteria. Neither dose of PD‐217,014 improved the percentage of participants reporting adequate relief of abdominal pain/discomfort compared with placebo, either using the Rome II‐defined total cohort or Rome II and IV IBS bowel habit sub‐types. There were similar observations for secondary endpoints, and no association between abdominal pain or anxiety levels at baseline with participant improvement. PD‐217,014 was generally well tolerated.

**Conclusion:**

This first large, dose‐ranging trial examining the efficacy of PD‐217,014 showed no significant efficacy in participants with IBS or bowel habit sub‐types, irrespective of their pain and anxiety levels.

## Graphical abstract

The novel α2δ ligand, PD‐217,014 shows no efficacy in irritable bowel syndrome (IBS). If earlier α2δ ligands do have efficacy in reducing abdominal pain in IBS, then this efficacy does not extend to all members of this pharmacological class, whether suffering with high levels of pain and/or anxiety or not.

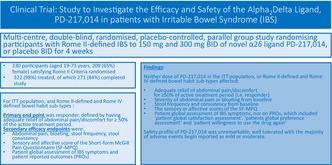

## 1 Introduction

Irritable bowel syndrome (IBS) is a disorder of gut–brain interaction, characterised by recurrent abdominal pain related to or relieved by defaecation, and associated with a change in the frequency and/or consistency of stool [1]. Prevalence varies globally from 5% to 20% [2], and the chronicity of symptoms has a significant impact on quality of life, along with work and social functioning [3]. Mood disorders also frequently co‐exist [4, 5].

Thus, IBS accounts for a considerable proportion of both secondary and tertiary referrals to gastroenterologists, with the presence and severity of abdominal pain usually being the main predictor of healthcare seeking [6, 7]. Traditional treatments of abdominal pain in IBS include antispasmodic agents and antidepressants, with tricyclic antidepressants ranking first for efficacy in a recent network meta‐analysis [8], although not significantly different from that of antispasmodics or peppermint oil, upon indirect comparison [8]. More recent pharmacological therapies for IBS patients with diarrhoea (IBS‐D) including alosetron, ramosetron, rifaximin and eluxadoline have all been shown to be superior to placebo according to the Food and Drug Administration (FDA)‐recommended endpoint for trials in IBS (i.e., composite endpoint of improvement in both pain and stool consistency); with alosetron 1 mg twice daily ranked first for efficacy, based on the FDA‐recommended endpoint and ramosetron 2.5 μg once daily ranked first for effect on abdominal pain [9]. Similarly, secretagogues for IBS with constipation (IBS‐C) including linaclotide, lubiprostone, plecanatide and tenapanor have all been shown to be superior to placebo, with linaclotide (290 μg once daily) ranked first for efficacy based on the FDA endpoint, abdominal pain and complete spontaneous bowel movements [10]. However, treatments continue to have limited efficacy in some patients, particularly against abdominal pain, and thus there remains an unmet medical need for new effective pharmacological treatments of abdominal pain in IBS.

Ligands that bind to the α_2_δ auxiliary protein of voltage‐gated calcium channels expressed on afferent neurons in the nervous system, such as gabapentin and pregabalin, have been shown to modulate sensory transmission and to ameliorate pain in a wide range of animal models and in man [11]. Pregabalin, which is approved for the treatment of neuropathic pain associated with diabetic peripheral neuropathy as well as postherpetic neuralgia, has been shown to provide analgesic benefit in both chronic somatic and visceral pain syndromes, such as fibromyalgia and pancreatitis [11]. Pregabalin has also been used to treat generalised anxiety disorder [12] and, although not approved for use in IBS, has been shown to reduce gut visceral sensitivity in patients with IBS‐D and concomitant visceral hyper‐sensitivity [13] but not IBS patients with IBS‐C [14], although the latter observation maybe due to the low prevalence of visceral hyper‐sensitivity in IBS‐C. Moreover, the authors of the latter study suggested that a longer treatment duration and escalating doses of pregabalin may be required in order to achieve benefit with pregabalin for outcomes related to abdominal pain in IBS [14]. One small randomised, placebo‐controlled, parallel group clinical trial in 85 patients with IBS has reported 12 weeks of treatment with pregabalin to improve the Bowel Symptom Scale (BSS) scores for pain or discomfort and the overall symptom severity, bloating and diarrhoea BSS scores, but not the constipation BSS scores [15]. Adequate relief of IBS symptoms and quality of life scores, however, were no difference between those treated with pregabalin and placebo controls. Larger clinical trials are therefore much needed to confirm the efficacy of α_2_δ ligands in IBS and/or sub‐types of IBS.

PD‐217,014 is a novel α_2_δ ligand that was developed as a potentially more potent successor to gabapentin and pregabalin and has been shown to be effective at inhibiting pain responses in two different animal models of visceral pain but to be inactive under normal conditions, and to have no effect on gastrointestinal transit in animal studies [16].

Here we report the findings of a Phase 2 multi‐centre, placebo‐controlled randomised clinical trial of PD‐217,014 in patients with IBS. The primary objective of this study was to assess the effect of oral doses of PD‐217,014 on relief of abdominal pain/discomfort. Secondary objectives were to assess the safety and tolerability of oral doses of PD‐217,014 and to assess the effect on intensity of abdominal pain/discomfort, patient's global assessment of IBS symptoms, stool frequency and consistency, abdominal bloating and patient‐reported outcomes.

## 2 Materials and Methods

The study was conducted from January 2004 to March 2005 in 40 centres in Australia, Belgium, France, Germany, Sweden and the United Kingdom (Table S1). An additional three centres were initiated but did not randomise any subjects. Institutional Review Boards and/or Independent Ethics Committee(s) at each investigational centre participating in the study approved the study. Participants provided written informed consent upon study entry. Details of the clinical trial are publicly accessible at clinicaltrials.gov, registration number NCT00139672.

### 2.1 Subject Population

Men and women aged 18–75 years, diagnosed with irritable bowel syndrome (IBS), as defined by the Rome II criteria [17], and who had normal examination of colon anatomy (assessed by colonoscopy or flexible sigmoidoscopy and barium enema) within the last 5 years, if aged ≥ 50 years old or with a family history of colorectal cancer, were eligible for the study. If rectal bleeding had occurred in the last year, a colonoscopy or flexible sigmoidoscopy and barium enema had to be performed before entry into the study. Additional exclusion criteria included the presence of biochemical abnormalities, the use of drugs which could interfere with safety or efficacy assessment of PD‐217,014, the concomitant use of gabapentin (NB pregabalin had not been granted marketing authorisation at time of study), the presence of organic gastrointestinal disease, major abdominal surgery (except appendectomy, cholecystectomy and hysterectomy), severe constipation (< 1 bowel movement per week), lactose intolerance, abnormal thyroid function (if not stabilised by a constant dose of thyroid hormones), abnormal 12‐lead electrocardiogram, major psychiatric disorder (Hospital Anxiety and Depression Scale (HADS) [18] score > 15 on either anxiety or depression), abuse of alcohol or other recreational or non‐therapeutic drugs, creatinine clearance ≤ 30 mL/min (estimated from serum creatinine, body weight, age and sex using the Cockcroft and Gault equation), and participants who were unlikely to follow the study protocol or who had other severe pain, which might impair the self‐assessment of their abdominal pain/discomfort due to IBS.

### 2.2 Study Design

This was a multi‐centre, double‐blind, randomised, placebo‐controlled, parallel‐group study with three treatment groups comparing 150 mg BID and 300 mg BID of orally administered PD‐217,014, and placebo BID in participants with IBS.

The study consisted of a 2‐week treatment‐free baseline period (from which Rome II IBS bowel habit sub‐types [17], and subsequently Rome IV IBS bowel habit sub‐types [19] were categorised), a 4‐week double‐blind treatment period and a withdrawal period. Participants attended five scheduled clinic visits: V0 at screening, V1 at randomisation, V2 after 1 week of treatment, V3 after 4 weeks of treatment, and V4 two weeks after the end of the treatment period. Participants taking prohibited medications completed a 7‐day washout period before the 2‐week treatment‐free baseline period. Rescue medications included loperamide, if the participant had diarrhoea for more than 2 days, bisacodyl if the participant had no bowel movement for more than 4 days and paracetamol if the participant experienced pain for more than 3 days. All rescue medications could not be used for more than two consecutive days over a period of a week, and for paracetamol, dose could not exceed 4 g/day.

### 2.3 Randomisation

Following a 2‐week treatment‐free baseline period, all participants who met the inclusion/exclusion criteria were randomised to receive PD‐217,014 150 mg or PD‐217,014 300 mg or matched placebo, twice daily for 4 weeks. Randomisation criteria included a daily abdominal pain/discomfort score of 4–8, inclusive (using a 0–10 numerical rating scale) recorded by the telephone interactive voice response system for a least 7 days (which need not be consecutive) over the 2‐week baseline period, and at least 10 days of daily abdominal pain/discomfort scores recorded during the 2‐week baseline period. Participants were assigned to study treatment in accordance with a central, blocked computer generated pseudo‐random code using the method of random permuted blocks. Study drug compliance was assessed for each participant using pill counts from returned bottles, and lack of compliance defined as missing three of more consecutive days of study medication.

All analyses were conducted using the intent‐to‐treat (ITT) population which consisted of all subjects randomised to treatment.

### 2.4 Data Collected and Efficacy Endpoints

The following data were collected either daily or weekly, as appropriate, during the baseline, treatment period and withdrawal period using a telephone interactive voice response system:

The primary endpoint was a responder, defined by having adequate relief of abdominal pain/discomfort for ≥ 50% of the active treatment period. Using a telephone interactive voice response system, participants were asked on a daily basis ‘Have you had adequate relief of your IBS pain/discomfort during the past 24 hrs?’

Secondary efficacy endpoints were

- *Daily pain rating score* assessed using an 11‐point scale ranging from 0 (no pain) to 10 (worst possible pain). The participants described how intense their abdominal pain/discomfort had been during the past 24 h by choosing the appropriate number between 0 and 10.
- *Patient global assessment of IBS symptoms*, a subject‐rated instrument that measured change in a participant's overall well‐being, and symptoms of abdominal discomfort, pain and altered bowel habit compared with pre‐study feelings. This was measured on a 5‐point scale: 1 (completely relieved), 2 (considerably relieved), 3 (somewhat relieved), 4 (unchanged) and 5 (worse) and was performed on a weekly basis.
- *Daily stool frequency* which was the number of bowel movements during the previous 24 h.
- *Daily stool consistency* obtained from a 6‐point numerical scale with which the subject described their stool consistency during the past 24 h: 0 (none), 1 (watery), 2 (loose), 3 (formed), 4 (hard) and 5 (very hard).
- *Daily bloating* assessed using a 6‐point scale every 24 h: 0 (none), 1 (very mild), 2 (mild), 3 (moderate), 4 (severe) and 5 (very severe).

Additional secondary efficacy endpoints recorded in the CRF included:

- *Short‐form McGill Pain Questionnaire* (*SF‐MPQ*) [20] which consisted of 15 descriptors; 11 representing the sensory dimension of pain experience and four representing the affective dimensions. Each descriptor was ranked by the subject on a 4‐point intensity scale and totalled for each dimension. This was completed at randomisation (visit 1) and at visit 3 at the end of the treatment period.
- *Hospital Anxiety and Depression Scale* (*HADS*) [18], a self‐reported scale used to screen for the presence of depressive disorder in non‐psychiatric populations. The HADS consists of seven questions each relating to anxiety and depression and was completed at the screening visit (visit 0).

A patient‐reported outcome (PRO) questionnaire (patient global satisfaction assessment, patient global preference assessment and an assessment of the patient's willingness to use the drug again) was also completed by all participants at visit 3 at the end of the treatment period. For patient global satisfaction assessment, the question ‘Overall, how satisfied are you with the medication, which you have received for your IBS condition since entering the study?’ was asked, with response scale: 1 (very satisfied), 2 (satisfied), 3 (neither satisfied or dissatisfied), 4 (dissatisfied) and 5 (very dissatisfied) completed. For patient global preference assessment, the question ‘Overall, do you prefer the medication, which you have been given for your IBS condition since entering this study to the treatment or treatments, which you received before this study?’ was asked, and response scale: 1 (I definitely prefer the medication that I am receiving now), 2 (I have a slight preference for medication that I am receiving now), 3 (I have no preference), 4 (I have a slight preference for my previous treatment) and 5 (I have a definite preference for my previous treatment) completed. For the assessment of the patient's willingness to use medication again, the question ‘If it were available to you, after this study would you be willing to continue to use the medication that you have received for your IBS condition since you entered the study?’ was asked, and response scale 1 (I would definitely want to use the medication again), 2 (I might want to use the medication again), 3 (I do not know), 4 (I might not want to use the medication again) and 5 (I definitely would not want to use the same medication again) completed.

### 2.5 Safety

All randomised participants in the intent‐to‐treat (ITT) population were evaluated for safety. Adverse events were recorded throughout the treatment period and up to 7 days after the final dose of study drug, using the conventions and terminology of MedDRA version 7.1.

### 2.6 Statistical Analysis

Analyses were primarily conducted using the intent‐to‐treat (ITT) population which consisted of all participants who were randomised and received treatment, and who were evaluable against Rome II and Rome IV criteria. One exception was the analysis of the primary efficacy endpoint, which was conducted on participants in the ITT population with any available post‐baseline data for the primary efficacy endpoint, comprising 308 participants and is termed the Responder Evaluable Population. All analyses use baseline and Week 4 data or data over the first 4 weeks of the dosing period. Week 4 values were calculated as the average of the last seven available daily records. Baseline was calculated as the average of the available data during the 14‐day period prior to randomisation. Change from baseline was set to zero for patients with baseline but no available post‐randomisation data, otherwise available data were used for all analyses without imputation due to a small fraction of missing data; tabular and graphical displays report the number of participants with data included in the analyses. The primary endpoint was a binary variable that took a value of 1 if the participant had answered the question ‘Have you had adequate relief of your IBS pain/discomfort during the past 24 hours?’ on any post‐baseline day and achieved adequate relief ≥ 50% of those days during the treatment period.

The original study was powered for the primary endpoint, that is, the number (percentage) of responders. The power was 80% for a one‐sided test at an alpha level of 0.10 assuming the response rate for participants on placebo was 40% and 55% for the participants on PD‐217,014, or an odds ratio of 1.83, with 100 participants per arm. The study was not powered for sub‐group analyses. Therefore, *p* values are considered descriptive and not used for formal hypothesis testing. However, we followed the original sequential testing approach and first looked at the contrast of the high‐dose group versus the low‐dose group, then the low dose versus placebo and finally the high dose versus the low dose, stopping at the first test where *p* > 0.1. All confidence intervals are reported as two‐sided 80% intervals. Analyses were performed in the R statistical and programming language [21].

The primary endpoint was analysed by logistic regression. A stepwise procedure was performed on the entire cohort to choose appropriate covariates for all subset analyses. The logistic regression models included the primary endpoint as the dependent variable, treatment and selected covariates as the dependent variable. A proportional odds model was used for the analysis of the Patient Global Assessment and patient‐reported outcomes.

Continuous change from baseline analyses fitted a linear model with change from baseline as the dependent variable and, treatment arm, baseline value and any additional covariates selected by stepwise‐selection as covariates [country, sex, average stool consistency at baseline, average stool frequency at baseline, HAD depression score at baseline, HAD anxiety score at baseline]. Least‐squares means calculated in the emmeans R library [22] reported for estimates and treatment contrasts.

## 3 Results

### 3.1 Study Population and Demography

**FIGURE 1:**
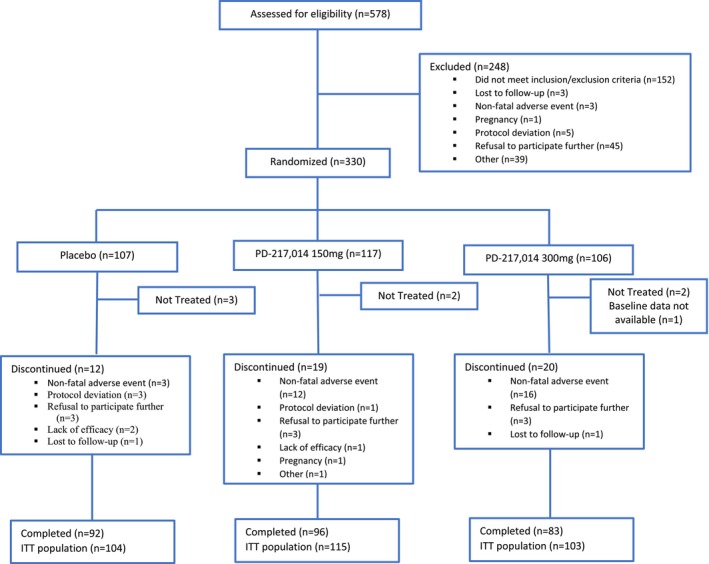
CONSORT flow diagram showing recruitment and patient disposition.

Of 578 participants with IBS screened for participation in the study, 330 (57%) entered into the 2‐week baseline period. Following this, all 330 participants were randomised to study treatment. Of the 330 participants randomised, 322 (98%) were treated, 7 (2%) were not treated, and 1 had no baseline data available; hence, these 322 participants were considered the ITT population, as previously stated. Of the treated participants, 271 (84%) completed the study and 51 (16%) discontinued from the study, 308 participants were available for the primary efficacy analysis. Figure 1 shows participant progression through the study for each treatment group.

Participant characteristics across the 2‐week treatment‐free baseline are shown in Table 1. The majority of participants were white and female, with an average age of 45.7 (range 19–73) years. Based on the Rome II criteria used to recruit patients, 164 participants (51%) were classified as IBS‐D, 81 (25%) IBS‐C and 77 (24%) IBS‐M. Subsequently, in a more recent analysis in which baseline data were used to identify participants satisfying the Rome IV criteria,164 participants (51%) were classified as IBS‐D, 65 (20%) IBS‐C, 36 (11%) IBS‐M and 56 (17%) as IBS‐U. One participant, for whom some efficacy data were also missing, could not be assigned to any Rome IV sub‐group with confidence and so was excluded from analyses. The baseline IBS characteristics of the ITT population and time since the first diagnosis of IBS were similar across treatment groups (Table 1). Baseline characteristics were generally similar across treatment groups for both Rome II‐ and Rome IV‐defined IBS bowel habit sub‐types (Tables S2 and S3).

**TABLE 1:** Demographic and baseline characteristics of subjects (total cohort) in the ITT population.

	Placebo (*n* = 104)	PD‐217,014 150 mg (*n* = 115)	PD‐217,014 300 mg (*n* = 103)	*p*
Sex				
Male	34 (33%)	42 (37%)	37 (36%)	0.82
Female	70 (67%)	73 (63%)	66 (64%)	
^a^Age (year)	43.2 (19–69)	47.9 (19–73)	45.8 (21–72)	0.04
Race				0.22
White	101 (97%)	114 (99%)	101 (98%)	
Black	1 (1%)	0	2 (2%)	
Asian	2 (2%)	0	0	
Other	0	1 (1%)	0	
IBS sub‐type (Rome II), *n* (%):				0.70
IBS‐D	51 (49%)	63 (55%)	50 (49%)	
IBS‐C	30 (29%)	24 (21%)	27 (26%)	
IBS‐M	23 (22%)	28 (24%)	26 (25%)	
IBS sub‐type (Rome IV), *n* (%)				0.45
IBS‐D	55 (53%)	65 (56.5%)	44 (43%)	
IBS‐C	22 (21%)	19 (16.5%)	24 (23%)	
IBS‐M	13 (13%)	11 (10%)	12 (12%)	
IBS‐U	14 (13%)	20 (17%)	22 (22%)	
Baseline IBS characteristics (total cohort)				
^a^ *Duration since first diagnosis of IBS* (*years*)	6 (0–41)	6 (0–35)	7 (0–48)	0.52
^b^Pain severity	5.10 (1.20)	5.19 (1.22)	5.11 (1.13)	0.86
^b^Bowel frequency	2.07 (1.32)	2.15 (1.36)	2.38 (1.76)	0.36
^b^Bowel consistency	2.79 (0.64)	2.66 (0.64)	2.83 (0.67)	0.14
^b^Bloating severity	3.26 (0.75)	3.18 (0.75)	3.19 (0.74)	0.69
McGill pain data				
^b^Sensory dimension	12.5 (6.02)	10.9 (5.84)	12.0 (6.40)	0.12
^b^Affective dimension	4.38 (3.20)	4.03 (2.99)	4.12 (3.47)	0.70
^b^HAD, anxiety	6.83 (3.51)	7.23 (3.67)	6.66 (3.68)	0.50
^b^HAD, depression	4.19 (3.03)	4.02 (2.82)	4.24 (3.29)	0.84

*Note:* Date expressed as either ^a^mean (range) or ^b^mean (SD).

### 3.2 Primary Outcome

**FIGURE 2:**
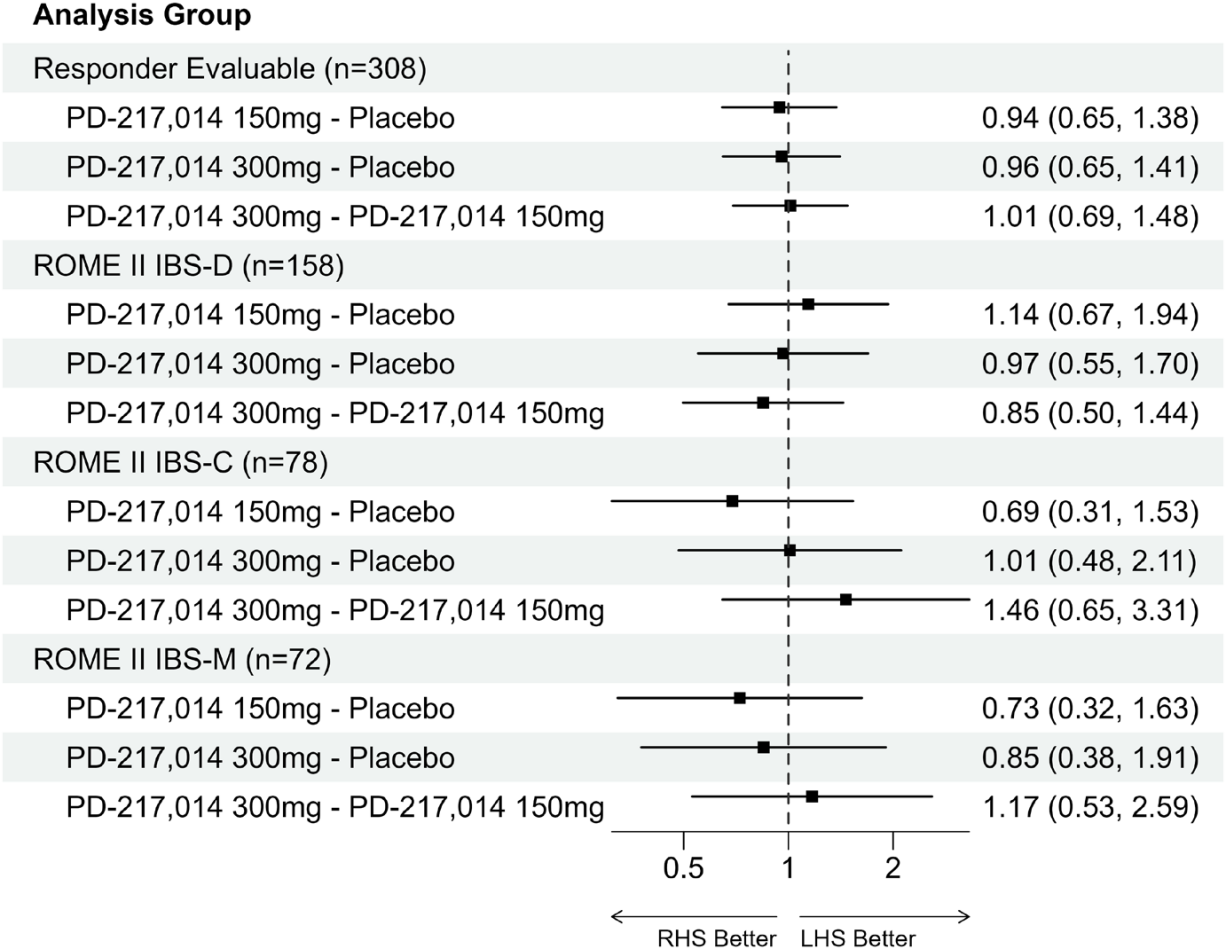
Forest plot for the primary endpoint, of ‘Responder’ in the Responder Evaluable Population, and in the participants in Rome II‐defined IBS bowel habit sub‐groups. The plots show the odds ratios (80% CI) of three comparisons (150 mg vs. placebo, 300 mg vs. placebo, and 300 mg vs. 150 mg). The *x*‐axis is labelled with the odds ratios on a logarithmic scale. An odds ratio of 1 indicates equality between groups. Numbers < 1 indicate that the treatment in the right‐hand side (RHS) of the descriptor is superior, whereas numbers > 1 indicate that the treatment in the left‐hand side (LHS) is superior.

Adequate relief of abdominal pain/discomfort for ≥ 50% of the active treatment period (i.e., responder) was not significantly different between the two doses of PD‐217,014 and placebo. Specifically, in the Responder Evaluable Population, responder rates (%) were 36.7% (36/98), 33.9% (38/112) and 35.7% (35/98) for placebo, 150 and 300 mg, respectively. Similar findings were made in the Rome II criteria‐defined IBS bowel habit sub‐types (Figure 2) and in the Rome IV‐defined IBS bowel habit sub‐types (Figure S1).

### 3.3 Secondary Outcomes

#### 3.3.1 Abdominal Pain, Bloating and Bowel Habit

**FIGURE 3:**
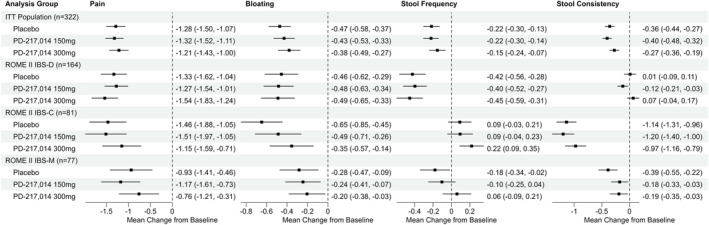
Forest plot showing mean change from baseline to Week 4 (80% CI) in the continuous secondary endpoints of abdominal pain, bloating, stool frequency and stool consistency in the ITT population, and in the participants in Rome II‐defined IBS bowel habit subgroups. Zero is the no‐effect reference.

**FIGURE 4:**
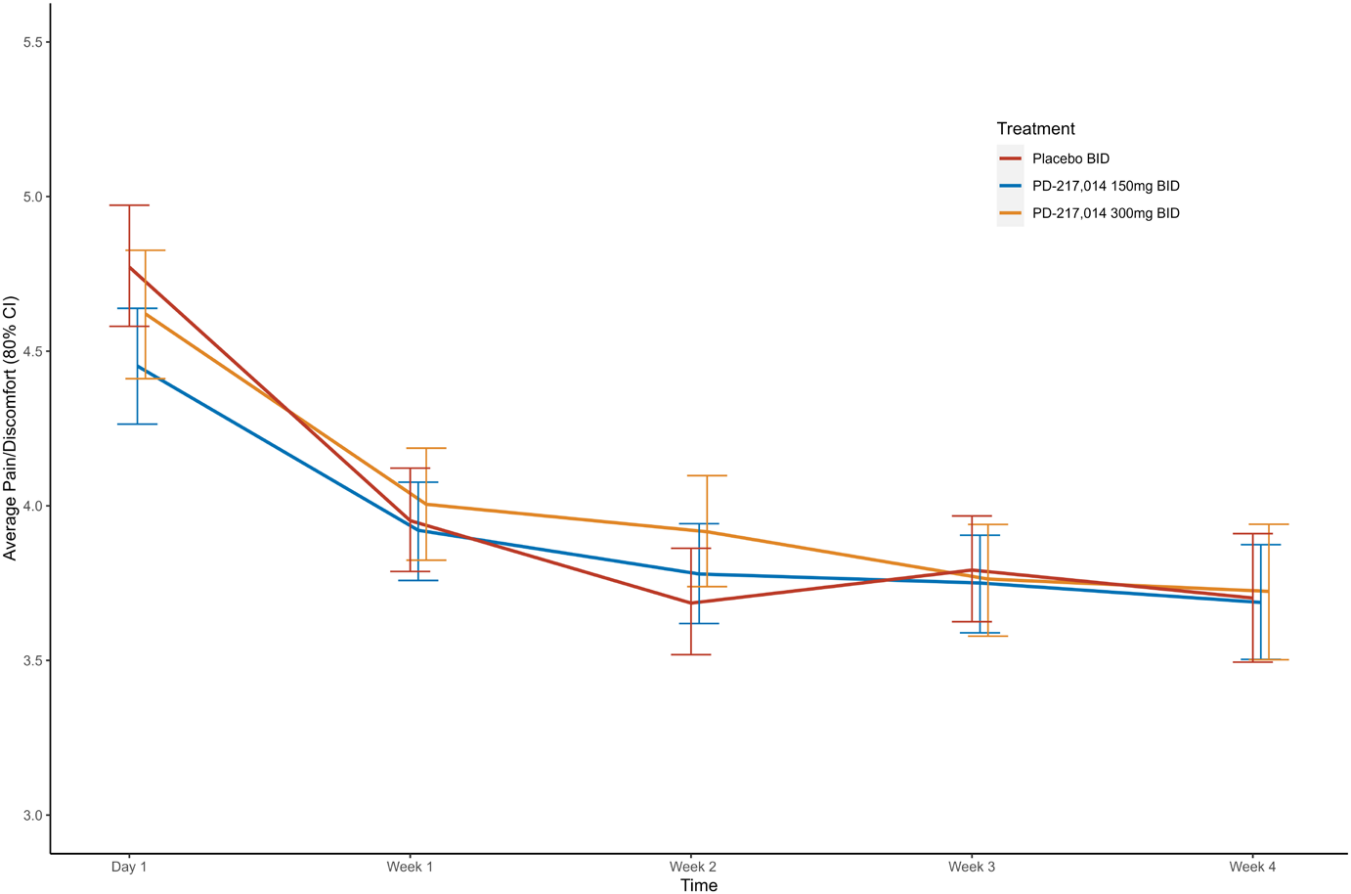
Change in average abdominal pain/discomfort (80% CI) across the 4 weeks of treatment with placebo (red), PD‐217,014 150mg BID (blue) and PD‐217,014 300mg BID (orange) in ITT population.

Neither dose of PD‐217,014 significantly changed the severity of abdominal pain or bloating from baseline in the ITT population at Week 4 or in sub‐groups of Rome II‐defined patients with IBS‐D, IBS‐C or IBS‐M, when compared with placebo (Figure 3) or when studied over time across the treatment period in the ITT population (Figure 4). Likewise, stool frequency and consistency were similarly unaltered (Figure 3). Analyses performed using the Rome IV‐defined IBS bowel habit subtypes were similar (Figure S2).

#### 3.3.2 Effect of Baseline Abdominal Pain, Depression and Anxiety

We examined whether baseline abdominal pain, anxiety or depression influenced the response to treatment, but we failed to find any statistically or clinically meaningful relationships. In summary, there was no evidence of an impact of these variables on the effect of PD‐217,014 on the primary endpoint. We did observe that greater anxiety and depression at baseline were associated with smaller decreases in abdominal pain scores over 4 weeks regardless of treatment arm, including placebo, that is they appeared to act independently of treatment.

#### 3.3.3 Short‐Form McGill Pain Questionnaire

**FIGURE 5:**
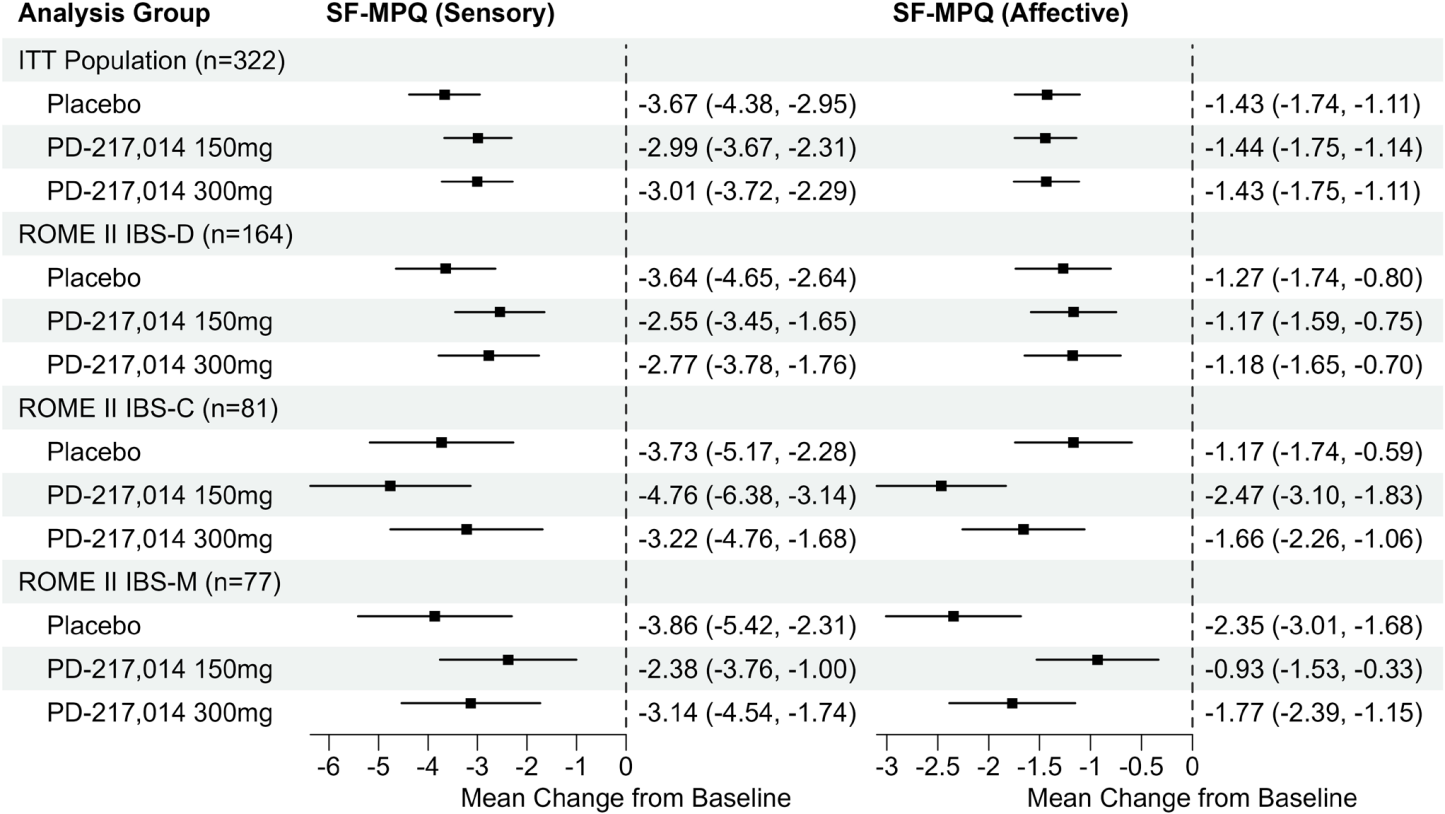
Forest plot showing mean change from baseline to Week 4 (80% CI) in the sensory and affective dimensions of pain experience using the short‐form McGill Pain Questionnaire (SF‐MPQ) in the ITT population, and in the participants in Rome II‐defined IBS bowel habit sub‐types. Zero is the no‐effect reference.

Compared to placebo, neither dose of PD‐217,014 significantly altered the sensory or affective scores of the short‐form McGill Pain Questionnaire in the ITT population or Rome II‐defined IBS sub‐groups (Figure 5). Analyses performed using the Rome IV‐defined IBS bowel habit sub‐types were similar (Figure S3).

#### 3.3.4 Patient Global Assessment of IBS Symptoms and Patient Reported Outcomes (PROs)

Compared to placebo, neither dose of PD‐217,014 showed consistent or significant effects on the patient global assessment of IBS symptoms (Figures S4 and S5). A post hoc responder analysis of patient global assessment of IBS symptoms was conducted with participants that had any symptom data and where a responder was defined as having symptoms considerably or completely relieved for 50% or more weeks. In this analysis, there was no difference between responder rates (%) for placebo, 150 and 300 mg PD‐217,014, and were 27.8% (27/97), 25.5% (27/106) and 26.1% (23/88), respectively.

Similarly, neither dose of PD‐217,014 showed consistent or significant effects on the PROs of ‘patient global satisfaction assessment’, ‘patient global preference assessment’ or ‘patient willingness to use the drug again assessment’, in either the Rome II‐defined sub‐types (Figure S4) or the Rome IV‐defined IBS sub‐types (Figure S5). A post hoc responder analysis of PRO responses was conducted for all participants for whom there were any data available and where a responder was defined as a participant who responded positively (1, very satisfied or 2, satisfied) to any of the PRO questions detailed in the Section 2. For ‘patient global satisfaction assessment’, there was no difference between responder rates for placebo (41.7%, 43/103), 150 mg (41.4%, 46/111) and 300 mg (49.0%, 48/98) PD ‐217,014. For ‘patient global preference assessment’, there was no difference between responder rates for placebo (47.1%, 48/102), 150 mg (40.5%, 45/111) and 300 mg (46.9%, 45/96) PD‐217,014. For ‘patient's willingness to use medication again assessment’, there was no difference between responder rates for placebo (59.2%, 61/103), 150 mg (53.2% 59/111) and 300 mg (56.1% 55/98) PD‐217,014.

#### 3.3.5 Safety

Overall, 219 participants (68%) experienced 494 treatment‐emergent adverse events (AEs) over 4 weeks of treatment with PD‐217,014. Of these events, 279 were mild, 192 moderate and 23 severe. AEs were reported by 70% of participants (*n* = 153 of 218) for PD‐217,014 and 64% (*n* = 67 of 104) for placebo. The most frequently reported all causality adverse events were headache, vertigo, dizziness, fatigue, abdominal pain and nasopharyngitis. One malignancy and no deaths were reported.

Five participants reported five serious adverse events that were considered to be treatment related by the investigator; three participants (one in the placebo group and two in the PD‐217,014 150 mg treatment group) had an increase in creatine phosphokinase, one subject in the PD‐217,014 300 mg treatment group had an asthenic reaction and possible withdrawal reaction, and another subject, also in the PD‐217,014 300 mg treatment group, had alcohol abuse. However, this event was not considered to be treatment related by the sponsor. There were also four serious adverse events that were not considered as treatment related, all of which occurred in the PD‐217,014 150 mg treatment group; pregnancy, bone pain, leiomyosarcoma and a small intestinal obstruction.

A total of 23 adverse events, reported by 18 (6%) participants were reported as severe adverse events. The incidence of severe adverse events was comparable in the placebo and PD‐217,014 150 mg treatment groups (four participants and five events and four participants and four events, respectively) but higher in the PD‐217,014 300 mg treatment group (10 participants with 14 events).

In total, 31 participants withdrew from the study due to treatment‐emergent adverse events; 3 in the placebo group, 12 in the PD‐217,014 150 mg treatment group and 16 in the PD‐217,014 300 mg treatment group. Three participants discontinued the study due to insufficient clinical response; two in the placebo group and one in the PD‐217,014 150 mg treatment group. The number of participants who were temporarily discontinued from the study or had their dose of study medication reduced was one in the placebo group, four in the PD‐217,014 150 mg treatment group and two in the PD‐217,014 300 mg treatment group.

The six system organ classes with the highest percentage of participants who experienced all‐cause adverse events were gastrointestinal disorders (*n* = 84, 26.1%), nervous system disorders (*n* = 83, 25.8%), infections and infestations (*n* = 52, 16.1%), general disorders and administration site conditions (*n* = 32, 9.9%), ear and labyrinth (*n* = 24, 7.5%), and musculoskeletal and connective tissue disorders (*n* = 20, 6.2%).

There were no clinically significant, treatment‐related changes, for the laboratory abnormalities. There were no clinically relevant changes in vital signs, ECG parameters and physical examination. The median change from baseline in supine blood pressure was similar across the treatment groups. There was little evidence of changes in QTcB or QTcF in any treatment group and no clinically relevant changes were observed, for the other ECG parameters.

## 4 Discussion

To date, there is very limited and underpowered data on the efficacy of α_2_δ ligands in the treatment of abdominal pain in patients with IBS, and thus a critical need for larger multi‐centre, double‐blind, randomised, placebo‐controlled trials to determine their true clinical utility. Using the novel α_2_δ ligand PD‐217,014, developed as a potentially more potent successor of the earlier, approved α_2_δ ligands, gabapentin and pregabalin, we have shown that 4 weeks treatment with either 150 mg and 300 mg BID PD‐217,014 did not differentiate from placebo on providing adequate relief of abdominal pain/discomfort for ≥ 50% of treatment time, nor on the severity of abdominal pain and bloating, or stool frequency and consistency. Moreover, sub‐grouping the IBS patients into the various bowel habit sub‐types meeting either Rome II or IV criteria prior to randomisation, again showed no benefit over placebo of either dose in any of the sub‐types.

Our observations contrast with those of Saito et al. [15] who reported that 12 weeks of treatment with the α_2_δ ligand, pregabalin (225 mg twice daily) improved pain/discomfort, bloating, diarrhoea and overall symptom severity scores in Rome III‐defined IBS patients. Like in our study, however, Saito et al. [15] showed no significant effect of pregabalin treatment on adequate relief of IBS symptoms, constipation severity or quality of life. In the Saito study, analysis of IBS sub‐types showed trends in improvement of abdominal pain/discomfort and overall IBS severity scores in patients with IBS‐D. These trends were also observed in the IBS‐M sub‐group, together with a significantly greater proportion of these patients reporting a 30‐point decrease in pain scores. However, as in our study with PD‐217,014, no effect of pregabalin was seen in patients with IBS‐C.

Possible explanations for differences between these two trials include duration of treatment, difference in patient cohorts enrolled (e.g., pathophysiology) and use of different Rome Criteria Classifications. Despite Saito et al. [15] collecting data weekly for 12 weeks, only scores over the last 4 weeks of treatment (i.e., weeks 9–12) were presented, and thus a direct comparison with data collected over 4 weeks of PD‐217,014 treatment cannot be made. The efficacy of pregabalin following the first 8 weeks of treatment was not reported, with the possibility that changes in IBS symptom severity and treatment‐related adverse events were different across these two different time periods, and possibly suggesting a slow onset of symptom relief for pregabalin. Neurological symptoms, such as dizziness and blurred vision, are well‐documented side effects associated with the use of α_2_δ ligands and were reported in both of these clinical studies. These adverse events generally diminish over the course of treatment, but whether treatment‐related adverse events in either clinical trial interfered with the reporting of gastrointestinal symptoms of IBS cannot be established. In our trial, a greater proportion of patients on placebo reported adverse events (64%) than those in the Saito et al. [15] trial (55%), particularly neurological symptoms (23% vs. 5%), whereas reporting of gastrointestinal side effects appeared similar across both trials. Another possible factor is the severity of IBS at the time of recruitment but examination of baseline abdominal pain scores, measured using a similar scoring system, appeared similar, despite the use of different Rome classifications to recruit patients. Abdominal pain severity has been shown to positively correlate with visceral sensitivity [23], which in a small study of patients with IBS with rectal hypersensitivity has been shown to be reduced by pregabalin [13]. Hypersensitivity to mechanical distension of the gut tends to be reported by more patients with IBS‐D than IBS‐C, while visceral hyposensitivity or insensitivity is seen more in patients with IBS‐C than IBS‐D, although for the latter at low percentages (i.e., < 20%) [24]. Whether gut hypersensitivity or indeed hyposensitivity in patients recruited affected one study cohort more than the other cannot be determined but similar percentages of patients with IBS‐D and IBS‐C were recruited by both trials, and the severity of baseline abdominal pain (which associates with visceral sensitivity) experience by patients in our study did not influence their response to PD‐217,014. α_2_δ ligands, such as pregabalin, also exhibit anxiolytic properties in both humans and animals, with pregabalin used to treat generalised anxiety disorder [12, 25, 26, 27]. α_2_δ ligands therefore might be expected to reduce pain, but if anything, patients in our study had slightly higher HAD anxiety scores than those in the Saito et al. trial [15]. Moreover, like pain severity, anxiety levels in our patient cohort did not appear to influence their response to PD‐217,014. Lastly, although there is limited data on PD‐217,014 compared with that for pregabalin, the effects of PD‐217,014 on visceral pain appear similar to those of pregabalin, with PD‐217,014 having been shown to be effective at inhibiting pain responses in several animal models of visceral, inflammatory and neuropathic pain but to have no effect on gastrointestinal transit (data on file at Pfizer) [16]. Despite no similar human studies, this supports its potential and that of other α_2_δ ligands as a treatment for abdominal pain in IBS and for more focused trials in IBS patients with visceral hypersensitivity. However, unfortunately, the development of PD‐217,014 is no longer active.

The safety profile of PD‐217,014 in this clinical study was unremarkable. Twice‐daily doses of 150 mg and 300 mg were generally well tolerated, with the majority of adverse events being reported as mild or moderate. The most common adverse events included those previously associated with the α_2_δ ligands gabapentin and pregabalin, including dizziness and somnolence.

Limitations of the current study include the lack of proven baseline hypersensitivity, the fact that Rome II criteria were used, although this was appropriate at the time of the trial, and applying the Rome IV criteria post hoc did not change our findings, and the lack of baseline classification of predominant bowel habit. However, these issues point to a potential lack of universal efficacy of α_2_δ ligands in the treatment of abdominal pain in IBS. The trial was also conducted between 2004 and 2005, the time at which the Rome II classification was used to define IBS but, as per the baseline 2‐week diary data, most fulfilled the Rome IV criteria for IBS, and it was felt by the authors that it was important that these findings of this trial were made available to the medical community. This study did not use the FDA‐recommended outcomes for IBS, as it was conceived and designed before the FDA guidance document was published in 2012 [28]. Thus, although we provide data on, for example, a reduction in pain from baseline, stool frequency and a measure of stool consistency, we cannot provide data on the latter as determined by the Bristol stool form scale.

To conclude, the lack of efficacy of PD‐217,014 in our study suggests that if earlier α_2_δ ligands do indeed have efficacy in reducing abdominal pain in IBS, then this efficacy does not extend to all members of this pharmacological class, whether suffering with high levels of pain and/or anxiety, or not.

## Author Contributions

**Lesley A. Houghton:** writing – original draft, supervision, project administration, formal analysis, writing – review and editing, data curation. **Simiao Gao:** formal analysis, writing – review and editing, data curation. **Steven A. Gilbert:** writing – review and editing, formal analysis, data curation. **Benoit Coffin:** investigation, writing – review and editing. **Magnus Simren:** investigation, writing – review and editing. **Jeremy D. Gale:** writing – review and editing, project administration, supervision, data curation, formal analysis.

## Disclosure

Lesley A. Houghton: has acted as a consultant for Pfizer, USA and served on scientific advisory boards for Ironwood Pharmaceuticals, USA; GSK, UK and Symprove UK. Simiao Gao: Pfizer employee. Steven A. Gilbert: Pfizer employee and stockholder. Benoit Coffin: is on the Board of Sanofi. Magnus Simren: Unrestricted research grants: Genetic Analysis AS; BioGaia, Consultant/Advisory Board member: Danone Nutricia Research; Biocodex; Tillotts; Takeda; Kyowa Kirin; Abbvie; BioGaia; Renapharma and Cinclus Pharma, Speakers' bureau: Tillotts; Kyowa Kirin; Takeda; Biocodex; Sanofi; Abbvie; Janssen Immunology; Pfizer; BioGaia; Renapharma; Mayoly and Bromatech. Jeremy D Gale: Pfizer employee and stockholder.

## Supporting information


**Figure S1.** Forest plot for the primary endpoint, of ‘Responder’ in the participants in Rome IV‐defined IBS bowel habit sub‐groups. The plots show the odds ratios (80% CI) of three comparisons (150 mg vs. placebo, 300 mg vs. placebo and 300 mg vs. 150 mg). The *x*‐axis is labelled with the odds ratios on a logarithmic scale. An odds ratio of 1 indicates equality between groups. Numbers < 1 indicate that the treatment in the right‐hand side (RHS) of the descriptor is superior, whereas numbers > 1 indicate that the treatment in the left‐hand side (LHS) is superior.

**Figure S2.** Forest plot showing the mean change from baseline to Week 4 (80% CI) in the continuous secondary endpoints of abdominal pain, bloating, stool frequency and stool consistency in the participants in Rome IV‐defined IBS bowel habit sub‐groups. Zero is the no‐effect reference.

**Figure S3.** Forest plot showing the mean change from baseline to Week 4 (80% CI) in the sensory and affective dimensions of pain experience using the short‐form McGill Pain Questionnaire (SF‐MPQ) in the participants in Rome IV defined IBS bowel habit sub‐types. Zero is the no‐effect reference.

**Figure S4.** Forest plot for the ordinal secondary endpoints of patient global assessment of IBS symptoms, and PROs in the ITT population and in the participants in Rome II‐defined IBS bowel habit sub‐groups, which included ‘patient global satisfaction assessment’, ‘patients global preference assessment’ and ‘patients willingness to use the drug again assessment’. The plots show the proportional odds ratios (80% CI) of three comparisons (150 mg vs. placebo, 300 mg vs. placebo and 300 mg vs. 150 mg). The *x*‐axis is labelled with the odds ratios on a logarithmic scale. An odds ratio of 1 indicates equality between groups. Numbers < 1 indicate that the treatment in the right‐hand side (RHS) of the descriptor is superior, whereas numbers > 1 indicate that the treatment in the left‐hand side (LHS) is superior.

**Figure S5.** Forest plot for the ordinal secondary endpoints of patient global assessment of IBS symptoms and PROs in the participants in Rome IV‐defined IBS bowel habit sub‐groups, which included ‘patient global satisfaction assessment’, ‘patients global preference assessment’ and ‘patients willingness to use the drug again assessment’. The plots show the proportional odds ratios (80% CI) of three comparisons (150 mg vs. placebo, 300 mg vs. placebo and 300 mg vs. 150 mg). The *x*‐axis shows the odds ratios on a logarithmic scale. An odds ratio of 1 indicates equality between groups. Numbers < 1 indicate that the treatment in the right‐hand side (RHS) of the descriptor is superior, whereas numbers > 1 indicate that the treatment in the left‐hand side (LHS) is superior.

**Table S1.** List of investigators.
**Table S2.** Demographic and baseline characteristics of subjects with IBS‐D, IBS‐C and IBS‐M as classified using the Rome II criteria in the ITT population.
**Table S3.** Demographic and baseline characteristics of subjects with IBS‐D, IBS‐C, IBS‐M and IBS‐U as classified using the Rome IV Criteria in the ITT population.

## Data Availability

Research data are not shared.
