# Supplementary figures and images for: Clinical Trial: Study to Investigate the Efficacy and Safety of the Alpha‐2‐Delta Ligand PD‐217,014 in Patients With Irritable Bowel Syndrome
Source: Aliment Pharmacol Ther. 2025 Jan 15;61(5):803–13. doi: 10.1111/apt.18487 (PMC11825930; doi:10.1111/apt.18487)

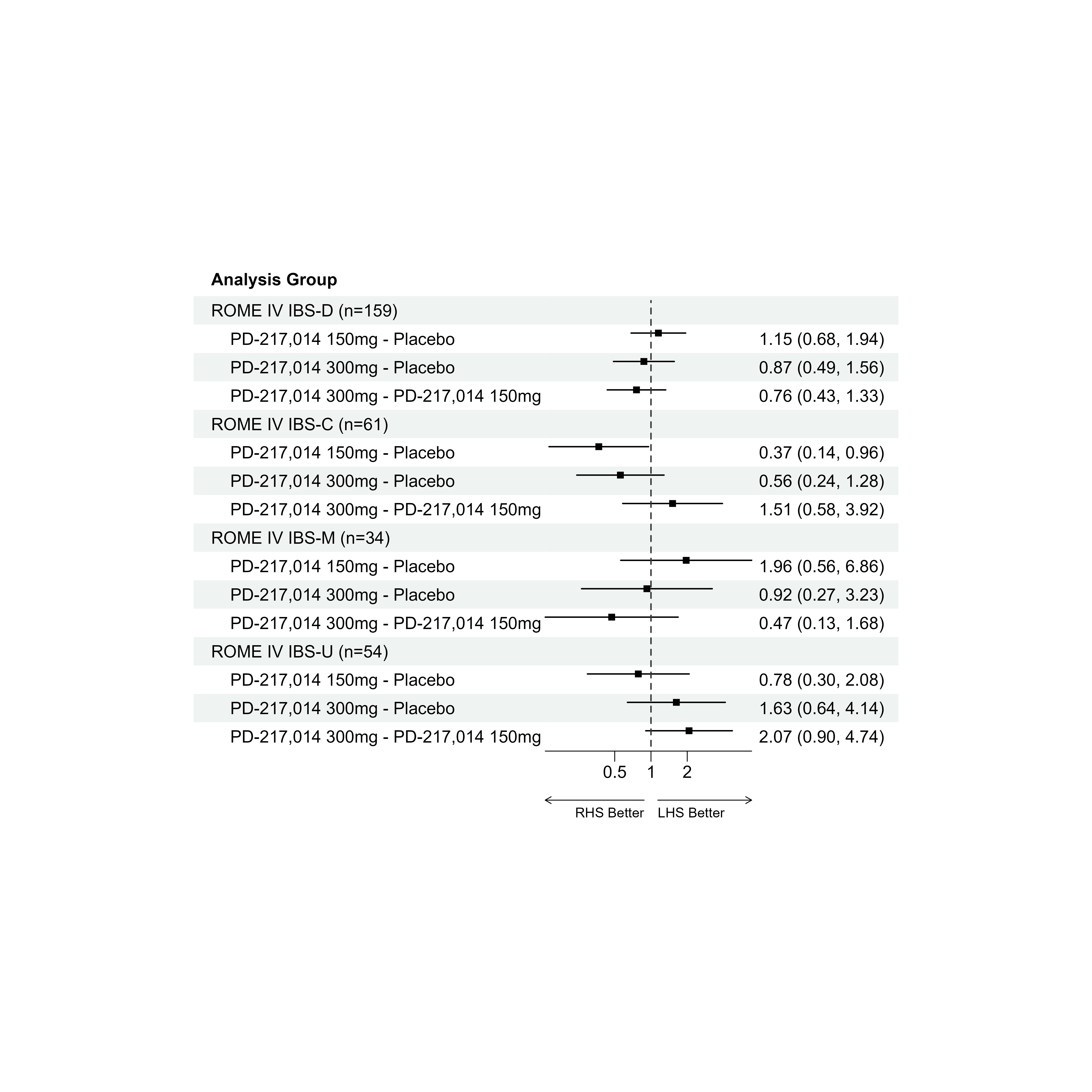

Supplement: Supplementary file 1 — Figure S1. Forest plot for the primary endpoint, of ‘Responder’ in the participants in Rome IV‐defined IBS bowel habit sub‐groups. The plots show the odds ratios (80% CI) of three comparisons (150 mg vs. placebo, 300 mg vs. placebo and 300 mg vs. 150 mg). The x‐axis is labelled with the odds ratios on a logarithmic scale. An odds ratio of 1 indicates equality between groups. Numbers < 1 indicate that the treatment in the right‐hand side (RHS) of the descriptor is superior, whereas numbers > 1 indicate that the treatment in the left‐hand side (LHS) is superior. [file APT-61-803-s004.png]

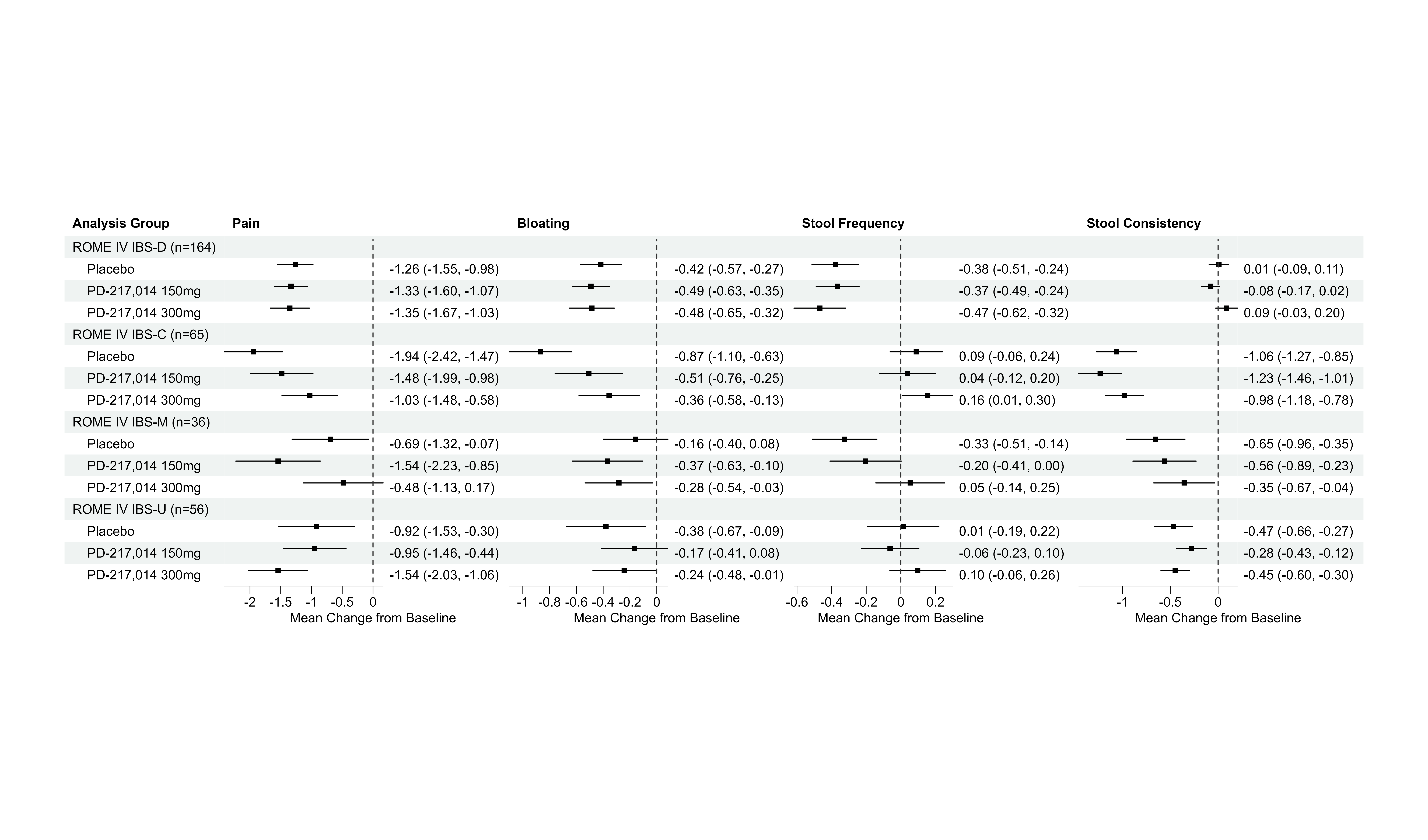

Supplement: Supplementary file 2 — Figure S2. Forest plot showing the mean change from baseline to Week 4 (80% CI) in the continuous secondary endpoints of abdominal pain, bloating, stool frequency and stool consistency in the participants in Rome IV‐defined IBS bowel habit sub‐groups. Zero is the no‐effect reference. [file APT-61-803-s006.png]

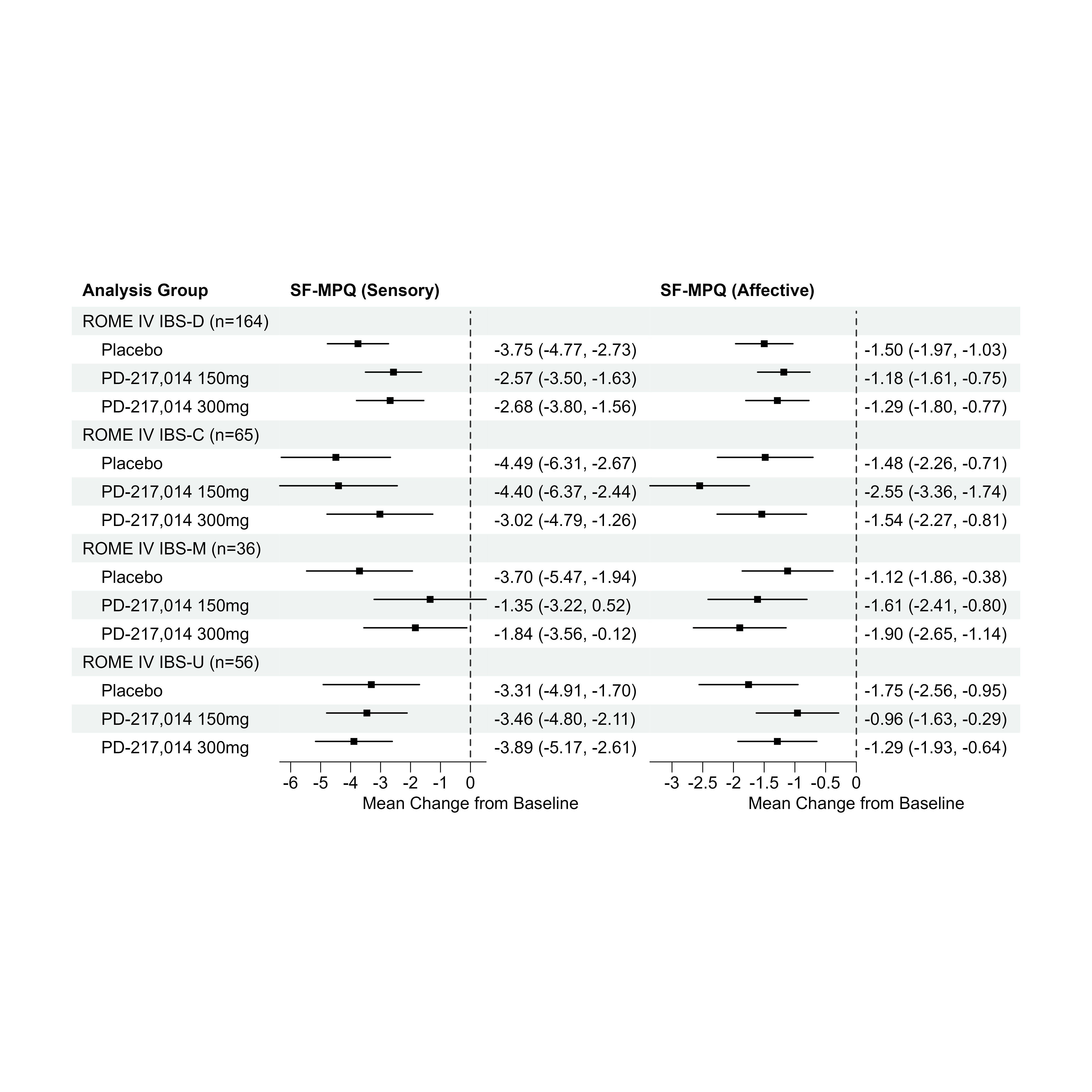

Supplement: Supplementary file 3 — Figure S3. Forest plot showing the mean change from baseline to Week 4 (80% CI) in the sensory and affective dimensions of pain experience using the short‐form McGill Pain Questionnaire (SF‐MPQ) in the participants in Rome IV defined IBS bowel habit sub‐types. Zero is the no‐effect reference. [file APT-61-803-s003.png]

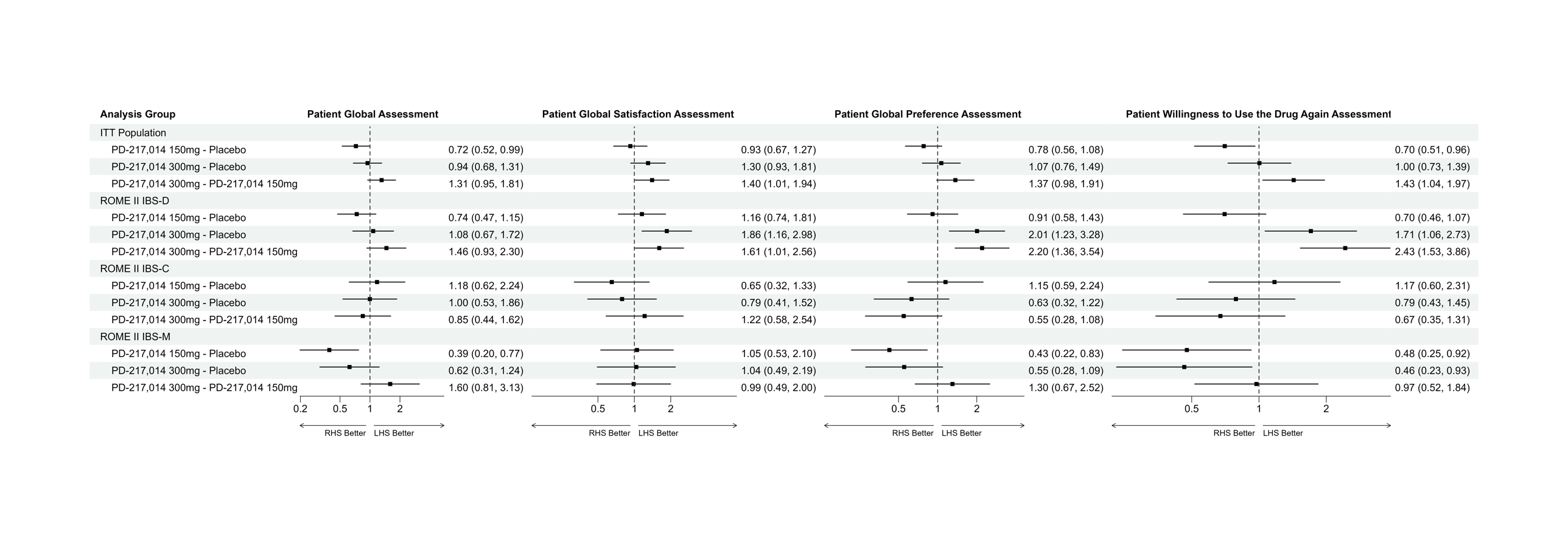

Supplement: Supplementary file 4 — Figure S4. Forest plot for the ordinal secondary endpoints of patient global assessment of IBS symptoms, and PROs in the ITT population and in the participants in Rome II‐defined IBS bowel habit sub‐groups, which included ‘patient global satisfaction assessment’, ‘patients global preference assessment’ and ‘patients willingness to use the drug again assessment’. The plots show the proportional odds ratios (80% CI) of three comparisons (150 mg vs. placebo, 300 mg vs. placebo and 300 mg vs. 150 mg). The x‐axis is labelled with the odds ratios on a logarithmic scale. An odds ratio of 1 indicates equality between groups. Numbers < 1 indicate that the treatment in the right‐hand side (RHS) of the descriptor is superior, whereas numbers > 1 indicate that the treatment in the left‐hand side (LHS) is superior. [file APT-61-803-s001.png]

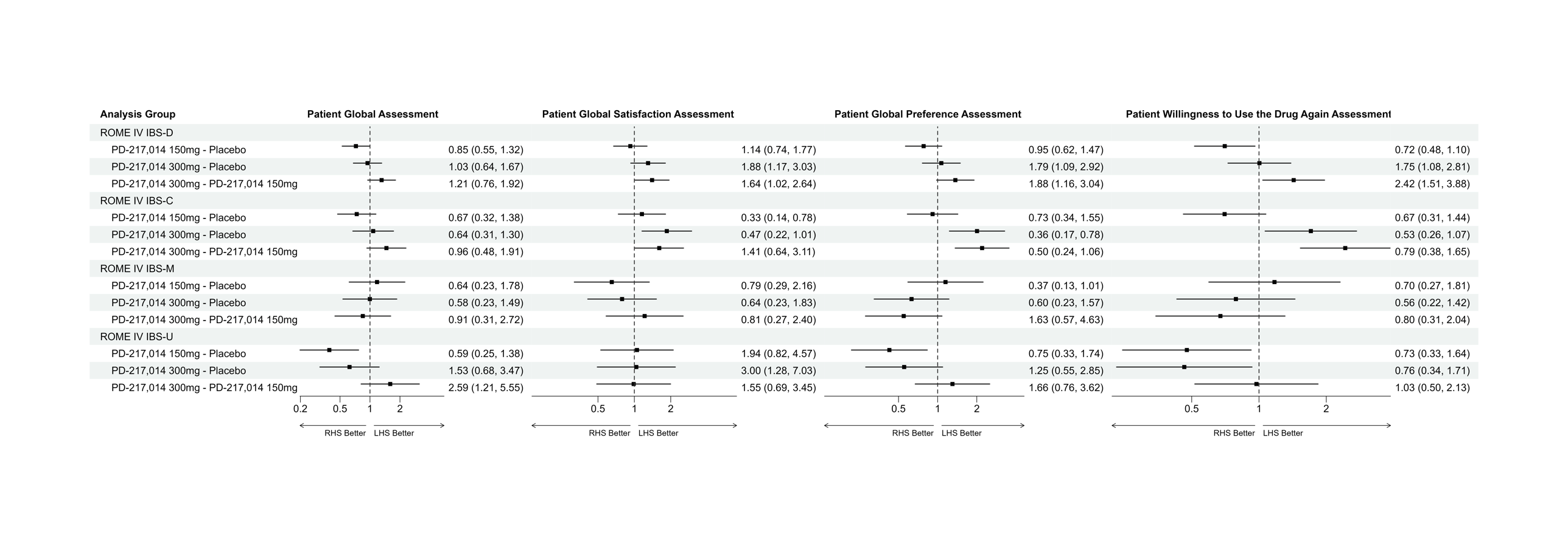

Supplement: Supplementary file 5 — Figure S5. Forest plot for the ordinal secondary endpoints of patient global assessment of IBS symptoms and PROs in the participants in Rome IV‐defined IBS bowel habit sub‐groups, which included ‘patient global satisfaction assessment’, ‘patients global preference assessment’ and ‘patients willingness to use the drug again assessment’. The plots show the proportional odds ratios (80% CI) of three comparisons (150 mg vs. placebo, 300 mg vs. placebo and 300 mg vs. 150 mg). The x‐axis shows the odds ratios on a logarithmic scale. An odds ratio of 1 indicates equality between groups. Numbers < 1 indicate that the treatment in the right‐hand side (RHS) of the descriptor is superior, whereas numbers > 1 indicate that the treatment in the left‐hand side (LHS) is superior. [file APT-61-803-s002.png]
