# Supplementary material for: Clinical Trial: Study to Investigate the Efficacy and Safety of the Alpha‐2‐Delta Ligand PD‐217,014 in Patients With Irritable Bowel Syndrome
Source: Aliment Pharmacol Ther. 2025 Jan 15;61(5):803–13. doi: 10.1111/apt.18487 (PMC11825930; doi:10.1111/apt.18487)
Supplement: Supplementary file 6 — Table S1. List of investigators. Table S2. Demographic and baseline characteristics of subjects with IBS‐D, IBS‐C and IBS‐M as classified using the Rome II criteria in the ITT population. Table S3. Demographic and baseline characteristics of subjects with IBS‐D, IBS‐C, IBS‐M and IBS‐U as classified using the Rome IV Criteria in the ITT population. [file APT-61-803-s005.docx]

**Supplementary Table 1:** List of investigators (*Did not randomise subjects)

| **Country** | **Site designation** | **Principal Investigator** |
| --- | --- | --- |
| Australia | 1001 | Agus E Brotodihardjo |
|  | 1002 | Sanjay Nandurkar |
|  | 1003 | Michael Gillman |
|  | 1004 | David J Hetzel |
|  | 1005 | John E Kellow |
|  | 1006 | Douglas Routley |
|  | 1007 | Alex Boussioutas and Neville D Yeomans |
|  | 1044 | Jeff Karrasch |
| Belgium | 1008 | Georges Coremans |
|  | 1009 | Bart D Schepper |
|  | 1010 | Erik Francois |
|  | 1011 | Fernand Fontaine |
|  | 1012 | *Daniel Urbain |
|  | 1013 | Danny De Looze |
|  | 1014 | *Marc Cabooter |
|  | 1033 | Hubert Piessevaux |
| France | 1016 | Michel Delvaux |
|  | 1018 | Phillipe Ducrotte |
|  | 1019 | Benoit Coffin |
|  | 1020 | Michel Dapoigny |
| Germany | 1026 | Rolf Roland Fink |
|  | 1027 | Thomas Simon |
|  | 1028 | Ulrich Frankenberger |
|  | 1029 | Eberhard Meier |
|  | 1030 | Thomas D Zoeller |
|  | 1031 | *Michael Karaus |
|  | 1032 | Hubert Moennikes |
|  | 1041 | Hans-Detlev Stahl |
|  | 1042 | Iris Henke |
|  | 1045 | Grzegorz Szelazek |
| Sweden | 1015 | Greger Lindberg |
|  | 1017 | Magnus Simrén |
|  | 1022 | Henry Nyhlin |
|  | 1023 | Per Hellstrom |
|  | 1024 | Ake Danielsson |
|  | 1047 | Carl-Peter Anderberg |
| United Kingdom | 1034 | John Randal Robinson |
|  | 1035 | Jean MK Fraser |
|  | 1036 | Jacqui M Maroni |
|  | 1037 | Hilary Shaw |
|  | 1038 | Nina Kola and Imran Lodhi |
|  | 1039 | Mansur Salman |
|  | 1043 | Richard M Langford |

**Supplementary Table 2: Demographic and baseline characteristics of subjects with IBS-D, IBS-C, IBS-M as classified using the Rome II Criteria in the ITT population**

| **IBS-D** | Placebo  (n=51) | PD-217,014  150 mg (n=63) | PD-217,014  300 mg (n=50) | P-value |
| --- | --- | --- | --- | --- |
| Male:Female  *Age (yr)  Baseline IBS characteristics:  *****Duration since 1^st^ diagnosis of IBS (yr)  ^†^Pain severity  ^†^Bowel frequency  ^†^Bowel consistency  ^†^Bloating severity  McGill Pain Data:  ^†^Sensory dimension  ^†^Affective dimension  ^†^HAD, Anxiety  ^†^HAD, Depression | 22:29  46.8 (19-69)  6 (0-34)  4.88 (1.15)  2.76 (1.42)  2.32 (0.34)  3.14 (0.68)  11.51 (5.72)  4.49 (3.16)  7.20 (3.24)  4.47 (2.89) | 27:36  48.5 (19-73)  6 (0-35)  5.01 (1.11)  2.68 (1.33)  2.28 (0.43)  3.06 (0.71)  10.84 (6.31)  3.65 (2.79)  6.89 (3.41)  3.76 (2.56) | 18:32  46.1 (21-71)  7 (0-48)  4.87 (1.10)  3.14 (1.90)  2.39 (0.45)  2.95 (0.72)  11.08 (6.12)  3.28 (3.03)  6.80 (3.66)  4.22 (3.06) | 0.70  0.63  0.62  0.75  0.34  0.37  0.42  0.84  0.14  0.82  0.37 |
| **IBS-C** | Placebo  (n=30) | PD-217,014  150 mg (n=24) | PD-217,014  300 mg (n=27) | P-value |
| Male:Female  *Age (yr)  Baseline IBS characteristics:  *****Duration since 1^st^ diagnosis of IBS (yr)  ^†^Pain severity  ^†^Bowel frequency  ^†^Bowel consistency  ^†^Bloating severity  McGill Pain Data:  ^†^Sensory dimension  ^†^Affective dimension  ^†^HAD, Anxiety  ^†^HAD, Depression | 6:24  43.6 (21-64)  8 (0-41)  5.56 (1.11)  1.24 (0.77)  3.50 (0.49)  3.42 (0.85)  12.17 (5.42)  3.50 (2.86)  6.40 (3.76)  4.07 (3.32) | 5:19  44.4 (22-72)  4 (0-12)  5.62 (1.41)  1.02 (0.50)  3.44 (0.49)  3.37 (0.65)  12.13 (6.25)  4.75 (3.38)  6.79 (3.73)  3.92 (2.93) | 8:19  44.7 (23-68)  8 (0-33)  5.41 (1.19)  0.97 (0.57)  3.59 (0.49)  3.49 (0.70)  14.44 (6.74)  4.93 (3.28)  6.70 (3.73)  4.07 (3.41) | 0.65  0.93  0.09  0.82  0.31  0.54  0.81  0.33  0.17  0.92  0.98 |
| **IBS-M** | Placebo  (n=23) | PD-217,014  150 mg (n=28) | PD-217,014  300 mg (n=26) | P-value |
| Male:Female  *Age (yr)  Baseline IBS characteristics:  *****Duration since 1^st^ diagnosis of IBS (yr)  ^†^Pain severity  ^†^Bowel frequency  ^†^Bowel consistency  ^†^Bloating severity  McGill Pain Data:  ^†^Sensory dimension  ^†^Affective dimension  ^†^HAD, Anxiety  ^†^HAD, Depression | 6:17  34.5 (21-67)  6 (0-20)  5.00 (1.32)  1.62 (0.72)  2.89 (0.40)  3.33 (0.72)  15.09 (6.85)  5.26 (3.53)  6.57 (3.81)  3.74 (3.00) | 10:18  49.6 (23-71)  6 (0-24)  5.21 (1.23)  1.90 (1.31)  2.87 (0.40)  3.30 (0.86)  9.89 (4.13)  4.25 (3.03)  8.36 (4.06)  4.68 (3.23) | 11:15  46.2 (24-72)  6 (0-34)  5.28 (1.08)  2.36 (1.38)  2.89 (0.40)  3.35 (0.68)  11.31 (6.14)  4.88 (4.10)  6.35 (3.78)  4.46 (3.70) | 0.49  <0.001  0.94  0.72  0.07  0.98  0.96  0.01  0.54  0.14  0.55 |

Date expressed as either *Mean (range) or ^†^mean (SD)

**Supplementary Table 3: Demographic and baseline characteristics of subjects with IBS-D, IBS-C, IBS-M, IBS-U as classified using the Rome IV Criteria in the ITT population**

|  | Placebo  (n=55) | PD-217,014 150 mg  (n=65) | PD-217,014 300 mg  (n=44) | P-value |
| --- | --- | --- | --- | --- |
| **IBS-D**  Male:Female  *Age (yr)  Baseline IBS characteristics:  *****Duration since 1^st^ diagnosis of IBS (yr)  ^†^Pain severity  ^†^Bowel frequency  ^†^Bowel consistency  ^†^Bloating severity  McGill Pain Data:  ^†^Sensory dimension  ^†^Affective dimension  ^†^HAD, Anxiety  ^†^HAD, Depression | 22:33  46.0 (21-69)  6 (0-34)  4.88 (1.20)  2.62 (1.46)  2.32 (0.33)  3.12 (0.72)  12.42 (6.29)  4.78 (3.29)  6.96 (3.22)  4.55 (3.04) | 29:36  48.3 (19-73)  6 (0-35)  5.09 (1.13)  2.51 (1.34)  2.25 (0.41)  3.05 (0.71)  10.88 (6.04)  3.98 (3.06)  7.11 (3.51)  3.86 (2.73) | 17:27  46.2 (21-71)  7 (0-48)  4.88 (1.12)  3.08 (2.02)  2.30 (0.43)  2.97 (0.69)  11.11 (6.03)  3.52 (2.97)  7.09 (3.65)  4.20 (3.33) | 0.79  0.68  0.53  0.27  0.53  0.59  0.37  0.13  0.97  0.44 |
|  | Placebo  (n=22) | PD-217,014 150 mg  (n=19) | PD-217,014 300 mg (n=24) | P-value |
| **IBS-C**  Male:Female  *Age (yr)  Baseline IBS characteristics:  *****Duration since 1^st^ diagnosis of IBS (yr)  ^†^Pain severity  ^†^Bowel frequency  ^†^Bowel consistency  ^†^Bloating severity  McGill Pain Data:  ^†^Sensory dimension  ^†^Affective dimension  ^†^HAD, Anxiety  ^†^HAD, Depression | 5:17  42.5 (21-61)  9 (0-41)  5.59 (1.00)  1.43 (0.80)  3.69 (0.38)  3.43 (0.91)  12.45 (5.76)  3.23 (2.39)  6.77 (3.90)  3.95 (3.14) | 1:18  43.1 (22-65)  5 (0-12)  5.34 (1.52)  1.24 (0.94)  3.63 (0.40)  3.38 (0.64)  12.32 (6.94)  5.26 (3.21)  7.26 (4.04)  4.11 (2.88) | 6:18  46.3 (23-68)  7 (0-33)  5.24 (1.15)  1.29 (1.03)  3.68 (0.47)  3.53 (0.68)  15.21 (5.87)  5.42 (3.19)  6.29 (3.29)  3.62 (2.93) | 0.21  0.52  0.18  0.55  0.78  0.89  0.74  0.21  0.02  0.70  0.86 |
|  | Placebo  (n=13) | PD-217,014 150 mg (n=11) | PD-217,014 300 mg (n=12) | P-value |
| **IBS-M**  Male:Female  *Age (yr)  Baseline IBS characteristics:  *****Duration since 1^st^ diagnosis of IBS (yr)  ^†^Pain severity  ^†^Bowel frequency  ^†^Bowel consistency  ^†^Bloating severity  McGill Pain Data:  ^†^Sensory dimension  ^†^Affective dimension  ^†^HAD, Anxiety  ^†^HAD, Depression | 3:10  35.8 (21-65)  8 (0-19)  5.70 (1.34)  1.71 (0.88)  3.07 (0.40)  3.58 (0.50)  15.23 (6.88)  5.08 (4.03)  6.62 (4.35)  3.54 (2.88) | 3:8  50.6 (39-63)  4 (0-12)  5.38 (1.05)  1.61 (0.92)  2.86 (0.28)  3.66 (0.48)  8.45 (4.27)  3.09 (2.55)  8.55 (4.20)  4.55 (2.46) | 8:4  41.3 (26-64)  6 (0-24)  5.57 (1.14)  2.36 (1.77)  2.88 (0.37)  3.42 (0.68)  10.50 (5.57)  4.33 (3.73)  6.33 (4.25)  4.25 (3.39) | 0.053  0.005  0.31  0.81  0.45  0.31  0.62  0.03  0.33  0.42  0.66 |
|  | Placebo  (n=14) | PD-217,014 150 mg (n=20) | PD-217,014 300 mg (n=22) | P-value |
| **IBS-U**  Male:Female  *Age (yr)  Baseline IBS characteristics:  *****Duration since 1^st^ diagnosis of IBS (yr)  ^†^Pain severity  ^†^Bowel frequency  ^†^Bowel consistency  ^†^Bloating severity  McGill Pain Data:  ^†^Sensory dimension  ^†^Affective dimension  ^†^HAD, Anxiety  ^†^HAD, Depression | 4:10  40.1 (19-67)  4 (0-10)  4.65 (1.04)  1.26 (0.64)  2.93 (0.26)  3.27 (0.68)  10.29 (3.54)  3.93 (2.87)  6.57 (3.48)  3.79 (3.09) | 9:11  49.8 (23-71)  6 (0-24)  5.24 (1.35)  2.12 (1.53)  3.00 (0.18)  3.14 (0.94)  10.85 (4.61)  3.50 (2.54)  6.85 (3.65)  4.15 (3.34) | 6:16  46.9 (24-72)  7 (0-34)  5.10 (1.04)  2.16 (1.21)  2.98 (0.17)  3.09 (0.75)  10.91 (7.26)  3.91 (4.29)  6.36 (4.05)  5.05 (3.64) | 0.43  0.14  0.28  0.32  0.01  0.69  0.74  0.90  0.88  0.92  0.53 |

Date expressed as either *Mean (range) or ^†^mean (SD)
